# Regulating China's health code system to prepare for future pandemics

**DOI:** 10.3389/fpubh.2023.1208615

**Published:** 2023-09-29

**Authors:** Taixia Shen, Chao Wang

**Affiliations:** ^1^Law School & Intellectual Property School, Jinan University, Guangzhou, China; ^2^Faculty of Law, University of Macau, Taipa, Macau, China

**Keywords:** China's health code system, future pandemics, rule of law, human rights, algorithmic governance, public health

## Abstract

This study investigates the challenges that China's health code system presents to individuals' lives and social development, using normative analysis and a case study. It looks for effective strategies to reform and regulate this system to prepare for future pandemics. Health code apps and mini programs have been widely deployed as effective tools for COVID-19 containment in China. However, their widespread and improper use has created risks due to the lack of both a systematic design and a basic supervision mechanism. The health code system risks infringing on individual privacy during data collection and storage. During the pandemic, the right to liberty and the right to treatment of Chinese citizens who lacked an appropriate health code were severely compromised. In some instances, the health code system was used as a stability maintenance tool by the authorities through arbitrary health code conversions. This article argues that China's health code systems should be reformed and regulated in preparation for future pandemics and that a new act regulating its management and use should be launched at the national level. Data collection, retention, and processing should be limited to the minimum amount of data needed to achieve the objective of protecting public health. The health code conversion power wielded by the authorities should be defined and regulated, the rules and procedures of code conversion should be transparent, arbitrary health code conversion behaviors should be prevented and punished, and persons whose rights have been violated by wrongful code conversion should have access to legal remedies.

## 1. Introduction

During the novel coronavirus pneumonia (COVID-19) pandemic, various levels of the Chinese government collaborated with high-tech companies to implement a health code system that would control the spread of the virus. This data-powered system controls and traces people's contacts by utilizing mobile phone positioning data to generate a quick medical response code that indicates an individual's health status. Health codes are dynamic codes accessed via mobile phone apps or mini-programs. They are automatically generated and checked by local government systems using both information from users' self-declarations and disease control-related big data. To obtain a code, applicants are required to visit the sign-up page of a relevant app or mini-program and provide personal information such as their name, national identity or passport number, and phone number. They are also required to report their travel history and any possible contact with confirmed or suspected COVID-19 patients within the past 14 days, as well as symptoms such as fever, fatigue, a dry cough, a stuffy nose, or a sore throat. Once the authorities verify this information, each user is assigned a quick-response code indicating their health status: red (high risk), amber (potentially infectious), or green (low risk) (1). This system gradually became one of the most useful and powerful digital tools available for epidemic prevention and control. In addition, China's everyday use of the health code system connected app users to data systems that synthesized social information from the authorities responsible for public health, public transportation, border control, and community governance, ultimately facilitating the nationwide restoration of economic and social order (2). However, the implementation of China's health code system had a profound impact on individuals' rights and lives. Everyone had to show their codes at shops, stations, parks, airports, libraries, and other public places. The movements of individuals without a green health code were restricted. The color of a person's health code could even determine their living situation. For instance, individuals assigned a red code were required to promptly arrange for transfer to an isolation hotel or to self-isolate at home for 14 days under China's COVID-19 prevention and control measures. Due to the widespread and improper use of this health code system, individual privacy and personal rights were greatly compromised.

At the end of November 2022, China's central government finally relented and began shifting away from its almost 3-year-long hyper-restrictive “zero-COVID-19” strategy. The release of 10 new measures to optimize China's COVID-19 response sharply decreased the number of situations in which the health codes were used. For instance, a green health code was no longer needed to enter shops, use public transportation, or travel to another city within China (3). With China's adjustments to its COVID-19 measures, the issue of whether the widely used health codes should be eliminated altogether has caused heated debate. Given the precedent that the system created for abuse and the risks it posed to personal rights, some experts have argued that the health code system should be abandoned. For instance, Wang (4) argued that the health code system should be completely withdrawn following the cessation of pandemic controls. However, other experts have suggested that the health code system should be transformed from a management tool into a service utility. For example, the system could be used as an identification credential for medical services such as appointments, registration, prescriptions, and payment (5).

This article explores the main challenges that China's health code system poses to personal rights and interests and examines whether this system should be withdrawn completely or reformed and regulated to prepare for future epidemics. The remainder of the paper is organized as follows. Section 2 examines how the health code system challenged individuals' fundamental rights and interests during the COVID-19 pandemic. Section 3 discusses whether the system should be abandoned completely. Section 4 argues that the health code system should be regulated and normalized to prepare for future pandemics, and Section 5 draws conclusions based on the study's findings.

## 2. Challenges posed by China's health code system to individuals' rights and interests

### 2.1. Privacy issues in China's health code system

The health code system's challenges related to data security and personal information have been recognized by the central government. The Data Security Law (DSL) of the People's Republic of China (PRC) and the Personal Information Protection Law (PIPL) of the PRC were passed in 2021 to regulate the processes of collecting and using personal data and to protect personal privacy. These laws imposed stricter requirements for the protection of personal information during health code use than in the past.

Before the DSL and the PIPL, the Cyber Security Law (CSL) of the PRC, which was passed in December 2016, set rules for information collection and management. Article 5 of the PIPL establishes the principles of information use whereby the handling of personal information shall follow the principles of lawfulness, fairness, necessity, and good faith. Article 6 reaffirms that the collection of personal information shall be kept to a minimum scope and that the information shall be used for processing purposes; excessively collecting personal information shall not be allowed. However, there is no definition of the minimum scope of collecting personal information. In response to the outbreak of COVID-19, the government launched a health code system requiring users to provide their name, gender, mobile phone number, ID number, and other information. This system posed a great threat to personal privacy and information. Its main problems are highlighted in the following paragraphs.

First, consent is greatly devalued when personal information is collected to generate health codes. When registering with the system, users must click to agree to the system's user service agreement and privacy policy, which seems to indicate recognition of the principle of informed consent. In reality, however, the principle of informed consent is not upheld, as individuals cannot obtain a health code without consenting to the collection of personal information, causing them great inconvenience during travel.

Second, there is no effective safety management mechanism related to the system's control and storage of information. When information is disseminated through platforms such as WeChat and QQ, it is stored by commercial companies rather than in government databases, greatly compromising confidentiality and security. In such circumstances, information can flow rapidly, and when there are no identity-related safeguards pertaining to users viewing that information and no restrictions on the transfer of files between users, the information is more likely to leak than it would be otherwise (6). Reports have indicated that epidemic prevention and control departments at all levels in China unintentionally leaked and spread statistical personal privacy information during the COVID-19 pandemic (7). For example, there was an incident in which numerous celebrities' photos and ID information kept by the Beijing health code system were leaked and sold online (8).

In addition, personal location information is collected and provided to epidemic prevention and control departments, transportation providers, shopping malls, parks, schools, restaurants, and other public institutions. This means that the personal whereabouts of individuals are collected and recorded by the site code (which is one branch of the health code). The reason given for the collection of this information is that the health code system also incorporates civil aviation, railway, bus, and other traffic data; telecommunications operator data; and financial institution and payment data. Through data analysis, citizens' travel patterns can be determined and high-risk groups can be identified (9). As a result, the health code system became a powerful tool for tracking COVID-19 in China. Although this system improved administrative efficiency, its information collection and application processes create major risks (10).

Overall, there are many hidden privacy hazards in China's health code system due to its lack of institutional construction and its collection, storage, and disposal of massive amounts of basic and sensitive personal information.

### 2.2. The health code system and the right to liberty

Initially, the health code system was launched to facilitate citizens' rapid and safe movement during an emergent and abnormal period. In reality, the right to free movement greatly depends on the color of a person's health code. Different colors indicate different degrees of the right to liberty. People with green health codes enjoy a complete right to liberty, whereas people with red codes are required to immediately quarantine for 14 days, and their right to liberty is greatly restricted. People with yellow health codes cannot enter public places until their code color changes to green. Under the health code system, people without a green code are not allowed to enter public places, including parks, nor are they permitted to go to other countries, cities, or towns. Consequently, individuals' right to liberty is severely restricted by the health code system.

The sacrifice of some people's right to liberty in the interest of public health is somewhat acceptable to most Chinese people. However, both the absence of transparency in the coding rules and inaccurate coding violate individuals' right to liberty, along with other related rights and interests (11). Crucially, the right to liberty is the basis of other rights.

An extreme case of a rights violation that aroused public outrage was the “red code incident” in June 2022. Depositors of rural banks in Henan province, along with depositors in other provinces, such as Shandong, were assigned red health codes that prevented them from withdrawing their funds from Henan banks (12). According to statistics provided by the Zhengzhou Discipline Inspection Commission, 1,317 village and town bank depositors were assigned red codes in this incident. Of those depositors, 446 were assigned red codes when they entered Zhengzhou, and 871 who remained outside Zhengzhou were assigned red codes when they scanned a site code that others had sent to them (13).

### 2.3. The health code system and the right to treatment

The right to treatment is also closely connected with China's health code system. Theoretically, ordinary patients who were not infected with the SAR-CoV-2 virus should have enjoyed the right to timely and effective diagnosis, treatment, and rehabilitation during the epidemic prevention period. During the period of COVID-19 prevention and control, appropriate basic medical and health services should have been available and accessible. However, the ordinary patient's right to treatment was denied or interfered with if they did not have a green health code and had not received a negative nucleic acid certificate in the previous 24 or 48 hours. In one case, a woman in Xi'an had a miscarriage after being refused timely admission to Xi'an Gaoxin Hospital because her nucleic acid test results had expired (14).

Article 30 of the Regulation on the Administration of Medical Institutions of the PRC and Article 27 of the Law on Doctors of the PRC indicate that medical institutions must provide immediate treatment to critically ill patients. However, the Joint Prevention and Control Mechanism of the State Council released a notice on the technical guidelines for the prevention and control of novel coronavirus infection in medical institutions (third edition) on September 8, 2021. This notice claimed that medical institutions must make nucleic acid testing compulsory among key groups of newly hospitalized patients, accompanying persons, and staff (15). Based on this notice, local hospitals could only admit patients who had obtained a certificate of a negative result from the new coronavirus nucleic acid test no more than 24 or 48 hours prior to admission. As a result, the health code system restricted the right to treatment during the pandemic.

## 3. Should the health code system be abandoned?

Although it is obvious that the health code system has facilitated pandemic control and economic recovery in China, it has limitations. First, there have been problems involving the collection of huge amounts of personal data, including sensitive and basic private information. Second, the system has restricted individuals' right to liberty, right to treatment, and related rights and interests. In this context, the development of digital technologies may have strengthened authoritarian control by facilitating a wide range of human rights abuses (16). However, proposals to abolish the health code system across China are overly simplistic.

This article argues that China's health code system should be regulated and reformed rather than simply abolished. The primary bases for this argument are as follows.

First, the abolition of the health code system across China could cause wasted spending for local governments, at least to a degree. Local governments have spent a substantial amount of money on the establishment and normal running of the health code system. If China were to abolish the health code system, the need to establish new health codes in the event of future pandemics would incur further unnecessary costs for local governments.

**Figure 1:**
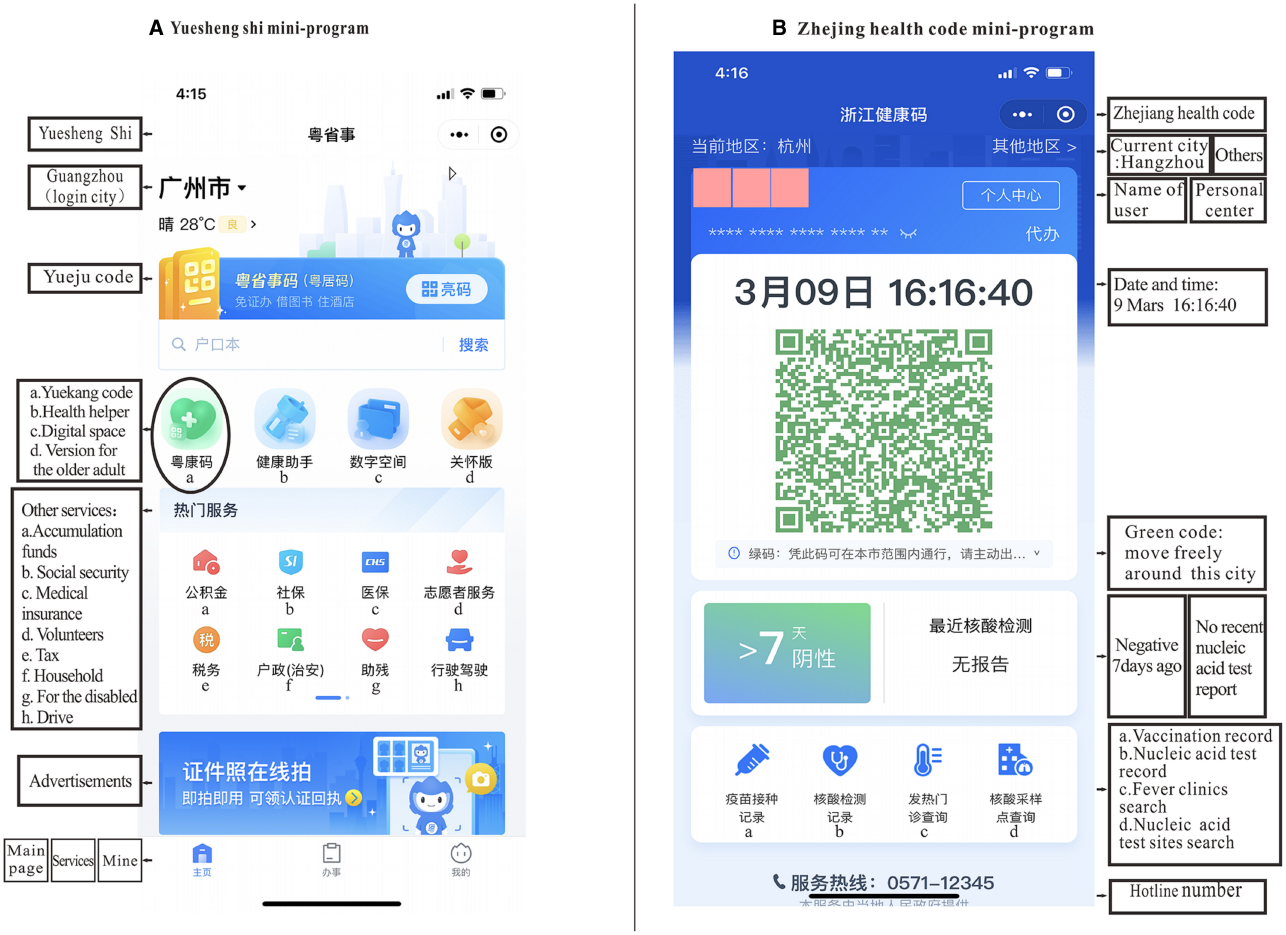
**(A)** Yuesheng Shi mini-program. **(B)** Zhejiang health code mini-program.

Second, the abolition of health codes across China could have negative effects on other government services and functions. All of the data related to the codes must be deleted or destroyed if the health code system is abandoned, according to the PIPL. However, different provinces use different platforms for the health code systems. In some provinces and cities, health code apps/mini-programs are embedded in apps/mini-programs used for other services, such as the Yuekang code (the health code of Guangdong province), which is embedded in in the Yuesheng Shi mini-program. Other provinces' health codes are separate mini-programs, such as the Zhejiang health code. Data stored on the first type of health code platform may be more difficult to delete than data stored on the second type of platform, as deleting health-code-related data in that situation may affect the operation of other government functions (17). Figure 1 shows the differences between the Yuekang code embedded in the Yuesheng Shi mini-program and the Zhejiang health code's mini-programs on WeChat.

**Figure 2:**
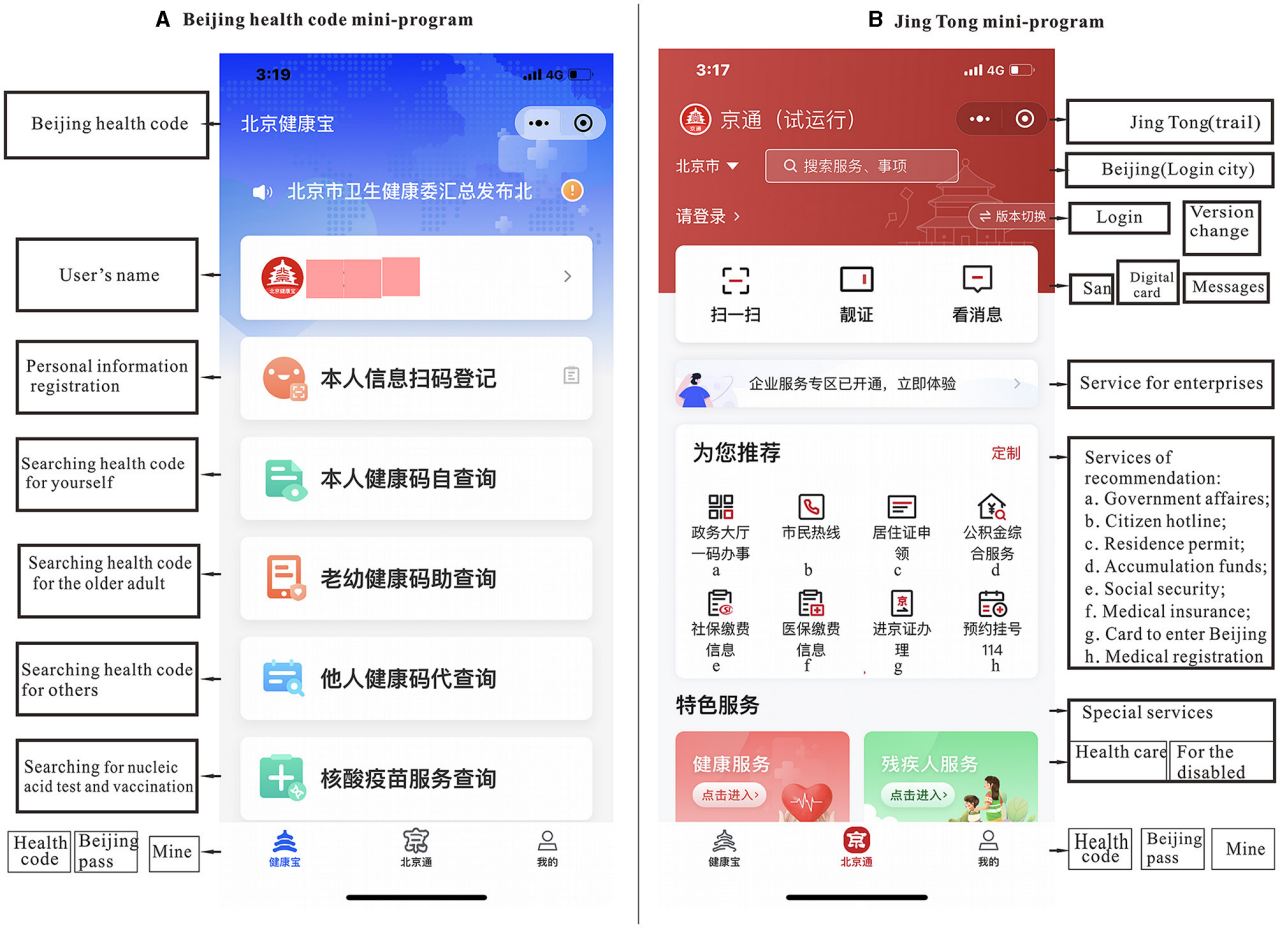
**(A)** Beijing health code. **(B)** Jingtong mini-program.

Third, it would be counterproductive to abolish the health code system, as it has completed a transformative upgrade in some cities and provinces. Some areas have added functions related to fever clinic inquiries, medical appointments, medication services, and transportation services to their original platforms, thus transforming the health code into a government service code. For instance, on December 19, 2022, Beijing launched its trial operation of the Jingtong mini-program, which integrated the “Beijing Pass” (a mini-program providing public services such as housing, accumulation funds, social security, and cards to enter Beijing) and the Beijing health code (Figure 2A). Citizens can choose whether to voluntarily extend the personal identity verification information they have provided under the Beijing health code to the Jingtong page. Figure 2B illustrates the integration of the Beijing Pass and the Beijing health code in the Jingtong mini-program. In addition, the Hainan provincial health code not only provides “one-code access” to Haikou Bus services but also connects users with a number of duty-free shops (18).

## 4. Can China's health code system be reformed to prepare for future pandemics?

During the COVID-19 pandemic, China's health code system has sacrificed some fundamental rights, such as the right to privacy, the right to liberty, and the right to treatment, on the grounds of protecting the public interest. The system's legal offenses and rights violations should be rectified, and the system should be regulated.

### 4.1. Legally upgrading the health code system for future pandemics

The travel history code was simultaneously removed from all platforms on December 13, 2022, and all traffic history and related data were also deleted (19). Several Yuekang code services, such as service portals for the older adult and young children, health declarations, and pandemic prevention workstations, recently closed, and all of the data collected for these services were taken offline and slated for destruction to protect individuals' personal information. However, the Yuekang code system remains operational (20). This is a good example of how to adjust the health code system consistent with the PIPL and national epidemic policy.

It is worth noting that in some provinces and cities, such as Hainan, Gansu and Beijing, local authorities have expanded the health code's functionality beyond pandemic control. The health code now encompasses scanning health codes, nucleic acid testing, and vaccination and governmental services, extending its functions to areas such as health care, transportation, and tourism. In a survey of adult health code app users aged 18 and above in the two major Chinese cities of Wuhan and Hangzhou, almost four out of 10 users supported the extensive use of health code apps, while the voices calling for restrictions or abolition were minimal (21). However, the issues of whether and how to upgrade or transform the health code system remain contentious in academic circles. Some scholars have argued that the health code system, as a special tool created in response to the unique needs and conditions of COVID-19 control, should be terminated. However, others have argued that the health code offers an important opportunity to upgrade both governance and public services in a post-pandemic society (22). One viewpoint suggests that the health code could be expanded by incorporating additional features such as online consultations, medical appointments and other public services. Another has proposed transforming the health code into a separate health care service code or public service code, thereby eliminating the original system (23). This article argues that any upgrade to the health code system should undergo scrutiny to ensure its compliance with the law. First, basic personal information such as names, IDs, and telephone numbers should be retained and protected by the health code system. However, information obtained from health declarations and COVID-19 prevention workstations should be deleted pursuant to the PIPL and related legislation on the information and data management field. Second, the algorithms and criteria used to determine an individual's health code should be legally and transparently established, as they greatly impact personal liberties and rights, such as the right to personal information, the right to privacy, and the right to know (24). Third, effective and accessible mechanisms should be implemented to allow individuals to appeal decisions and rectify errors or unfair assessments. These features would enhance the system's fairness, effectiveness, credibility, and respect for human rights and the rule of law.

Overall, the health code should be upgraded to a comprehensive code based on essential personal information, encompassing both the existing health code system and other public services like transportation, housing, and social security. However, personal information such as users' gender and age that will not be necessary for future pandemic control should be removed. If local governments decide to retain or update the health code system, it will be crucial to ensure their compliance with the relevant laws. Additionally, the new comprehensive code built upon the health code should not be mandatory, and thus individuals would have the freedom to choose whether to use it. In the event of future pandemics, it will be simple and convenient to widely utilize the upgraded health code.

### 4.2. Regulating the health code system at the national level

While retaining the health code system is a good way to prepare for a possible recurrence of the pandemic, considering the level of chaos during the process of creating local regulations on health codes, this article argues that the health code system should be regulated at a national level in future pandemics. The main reasons are set forth below.

First, current national laws regulating data and information use are still too vague to deal with the challenges posed by the health code system to personal privacy and other fundamental rights. Some Chinese laws, such as the CSL, the DSL, and the PIPL, have established principles of information use such as lawfulness, fairness, necessity, and good faith. Article 6 of the PIPL affirms that the collection of personal information shall be limited to the minimum scope necessary for processing and that the excessive collection of personal information shall not be allowed. However, it does not clearly define these terms or provide specific guidelines for defining the minimum necessary scope of collection or excessive collection. Article 19 of the PIPL stipulates that the retention period for personal information shall be the shortest time necessary to achieve the processing purpose, except as otherwise provided by any law or administrative regulation. However, it does not define or explain the shortest time necessary to achieve the processing purpose (25).

Furthermore, according to Article 8 (5) of the Legislation Law of the PRC, the health code system, which is intrinsically related to citizens' fundamental rights and freedoms, should be regulated by national laws. Nevertheless, the interim measures for the management of the health codes launched by central and local governments create the risk of excessive authority. In addition, the Interim Measures for the Management and Services of Health Codes for the Prevention and Control of the New Coronavirus Pneumonia Epidemic passed in January 2021, and local measures regulating the health code system suggested principled requirements for coding rules and data protection (26). However, there are no specific provisions addressing the rights and responsibilities related to health code management.

### 4.3. The main content of the health code system act for future pandemics

This article admits that the content and specific provisions of the health code bill or act of the PRC require clear and rigorous elucidation. They must at least address the following main points.

The PLPL provides no clear definition of either the minimum necessary scope or excessive collection related to the process of personal information collection. Thus, providing a clear scope of personal information collection for the upgraded health code system is a top priority.

Increasing the transparency of health code use and management rules is also vital. The vast majority of Chinese citizens have no idea where their health code-related information is stored or whether it is secure, and they do not know the details of the code conversion rules. Therefore, the law should regulate the operation and management of health codes, and the code conversion rules should be made transparent.

In addition, the laws should establish a relief system entitling the public to seek redressal from the relevant departments or judicial bodies if their rights have been violated through the misuse or misapplication of health codes.

## 5. Conclusions

Although China's health code system has aided the containment of COVID-19, it has also presented considerable challenges to the personal privacy, right to liberty, and right to treatment of its citizens due to the excessive collection, mishandling, and misuse of their personal data (24). In the long run, regulating China's health code system at the national level will help the nation to prepare for future pandemics. The process of health code application, information and data collection and storage rules, code conversion rules, and a relief system should be the main provisions of such legislation. Like all kinds of technologies, digital health codes systems have advantages and disadvantages and must be regulated. Humans and human rights should be placed at the center of pandemic prevention and control, and protecting and respecting those rights should be considered of primary importance.

## Data availability statement

The raw data supporting the conclusions of this article will be made available by the authors, without undue reservation.

## Author contributions

TS and CW contributed to the conception of the study and the writing, reviewing, and editing of the paper. All authors contributed to the manuscript revision and they read and approved the submitted version.
